# Modulation of Medium-Chain Fatty Acid Synthesis in *Synechococcus* sp. PCC 7002 by Replacing FabH with a *Chaetoceros* Ketoacyl-ACP Synthase

**DOI:** 10.3389/fpls.2016.00690

**Published:** 2016-05-26

**Authors:** Huiya Gu, Robert E. Jinkerson, Fiona K. Davies, Lyle A. Sisson, Philip E. Schneider, Matthew C. Posewitz

**Affiliations:** ^1^Department of Chemistry and Geochemistry, Colorado School of Mines, GoldenCO, USA; ^2^Department of Plant Biology, Carnegie Institution for Science, StanfordCA, USA

**Keywords:** medium chain fatty acids, *Chaetoceros*, type III ketoacyl-ACP synthase, *Synechococcus* sp. PCC 7002, lauric acid production, RNAseq

## Abstract

The isolation or engineering of algal cells synthesizing high levels of medium-chain fatty acids (MCFAs) is attractive to mitigate the high clouding point of longer chain fatty acids in algal based biodiesel. To develop a more informed understanding of MCFA synthesis in photosynthetic microorganisms, we isolated several algae from Great Salt Lake and screened this collection for MCFA accumulation to identify strains naturally accumulating high levels of MCFA. A diatom, *Chaetoceros* sp. GSL56, accumulated particularly high levels of C14 (up to 40%), with the majority of C14 fatty acids allocated in triacylglycerols. Using whole cell transcriptome sequencing and *de novo* assembly, putative genes encoding fatty acid synthesis enzymes were identified. Enzymes from this *Chaetoceros* sp. were expressed in the cyanobacterium *Synechococcus* sp. PCC 7002 to validate gene function and to determine whether eukaryotic enzymes putatively lacking bacterial evolutionary control mechanisms could be used to improve MCFA production in this promising production strain. Replacement of the *Synechococcus* 7002 native FabH with a *Chaetoceros* ketoacyl-ACP synthase III increased MCFA synthesis up to fivefold. The level of increase is dependent on promoter strength and culturing conditions.

## Introduction

Biologically derived diesel from water-oxidizing, photosynthetic microorganisms (PSMs) is considered an efficient and promising next-generation technology for the production of renewable fuels (Radakovits et al., 2010; Work et al., 2013). These photosynthetic organisms are capable of high photon conversion efficiencies and could be deployed so that their cultivation does not directly compete with the contemporary global food supply (Schenk et al., 2008; Mata et al., 2010). To improve commercial feasibility, research efforts have focused on areas ranging from optimizing photosynthetic yields to performing scalability assessments (Larkum et al., 2012; Ho et al., 2014; Quinn and Davis, 2015). These efforts can be combined with advances in next-generation DNA sequencing and metagenomic analysis to characterize the diversity of phototrophic life at the enzyme/molecular level, which facilitates the discovery of genetic “parts” that can be transformed into “chassis” organisms to genetically improve biotechnological phenotypes in production organisms (Kodzius and Gojobori, 2015).

While most contemporary research efforts are focused on improving biomass accumulation, relatively few efforts are targeting improved oil quality. However, lipid oil quality is a significant issue in diesel fuel utilization, as biological products typically have longer chain lengths and higher degrees of unsaturation relative to conventional petrodiesel (Durrett et al., 2008). These features lead to poor cold-flow temperature properties (longer chain lengths) and oxidative instability (higher unsaturation) (Knothe, 2005, 2008).

The genetic manipulation of fatty acid chain length in plants has succeeded in changing oil crops to synthesize more saturated, medium-chain fatty acids (MCFAs, C_8-14_), which are preferable for biodiesel (Voelker et al., 1992; Thelen and Ohlrogge, 2002). Expressing acyl-ACP thioesterases (acyl-ACP TEs) alone, or with ketoacyl-ACP synthase (KAS) from plants that produce MCFA, leads to MCFA accumulation in transgenic hosts (Jones et al., 1995; Leonard et al., 1998; Slabaugh et al., 1998). However, there are relatively few successful reports regarding MCFA accumulation in PSMs (Radakovits et al., 2011), and only limited information is available regarding mechanisms to increase MCFA synthesis in PSMs to produce a higher quality biofuel. Studies of TEs, the enzymes most commonly used to control fatty acid chain length, indicate differences between algal and plant TEs in terms of substrate recognition and phylogeny (Jing et al., 2011; Blatti et al., 2012; Beld et al., 2014).

In this study, we used bioprospecting, cell sorting, and fatty acid methyl ester (FAME) profiling to identify a diatom from Great Salt Lake (GSL), Utah (*Chaetoceros* GSL56 hereafter) that naturally accumulated high levels of C_14:0_ fatty acid (>16%), and initiated studies probing fatty acid synthesis (FAS) in this organism. We chose GSL as a sampling site because the growth of halophilic algae in high-salt production systems has the potential to yield strains that are productive in seawater raceways where evaporation leads to increased salt concentrations; and because unique enzymology of potential biotechnological relevance can be found in extremophiles isolated from hypersaline ecosystems (Meuser et al., 2013). *Chaetoceros* GSL56 accumulates among the highest levels of C_14:0_ in a PSM that we have observed to date. As an initial characterization of the MCFA accumulation phenotype, we probed the physiological parameters influencing C_14:0_ accumulation in this alga, and attained and assembled a whole-cell transcriptome to begin probing FAS enzymes. To validate gene annotations and to search for superior FAS enzymes, a targeted set of genes encoding eukaryotic FAS enzymes from this diatom were transformed into a cyanobacterium [*Synechococcus* sp. PCC 7002 (hereafter, *Synechococcus* 7002)] engineered to secrete fatty acids. We establish that a eukaryotic type III ketoacyl-ACP synthase (KASIII) enzyme can functionally replace the endogenous FAS enzyme FabH in *Synechococcus* 7002, and that expression of this non-native, eukaryotic enzyme improves MCFA yields under the culturing conditions used.

## Materials and Methods

### Strain Identification and Culturing

*Chaetoceros* GSL56 was isolated from Farmington Bay in Great Salt Lake (GSL), Utah, USA in 2008. Cultures were maintained and grown in f/2 medium, at 29°C in a Percival incubator (Percival, Perry, IA, USA) illuminated with white fluorescent light [∼40–120 μmol photons m^-2^ s^-1^ of photosynthetically active radiation (PAR)] using a 16/8 light/dark cycle. Cell numbers were measured using a Z2 Coulter Counter (Beckman-Coulter, Brea, CA, USA). The partial 18S rRNA gene (1162 bp) was amplified from genomic DNA and sequenced for taxonomic identification using the universal primers 360FE and 1492RE (Dawson and Pace, 2002). The 18S rRNA gene sequence was deposited in Genbank (accession no. HQ710801).

### FAME Quantification

Lipids were extracted and converted into FAMEs as described previously (Radakovits et al., 2012; Work et al., 2015) with some modifications. Briefly, 1.0 ml of methanol saturated with 5% KOH (0.8 g/ml) was added to 0.5 ml fresh culture samples in 4.0 ml sample vials, sealed and incubated at 100°C for 90 min resulting in cell lysis and lipid saponification. Acid-catalyzed methylation was then carried out by adding 1.5 ml 1:16 12N HCl/MeOH to the same vial and incubating at 80°C for 6 h. FAMEs were then extracted into 1.25 ml hexane via gentle inversion. Extracts were analyzed directly by gas chromatography-flame ionization detection (GC-FID) using an Agilent 7890A gas chromatograph equipped with a DB5-ms column (Agilent Technologies, Santa Clara, CA, USA). Different concentrations of the Supelco 37 component FAME standards mix (Supelco Inc., Bellefonte, PA, USA) were analyzed by GC-FID and used for peak identification and sample quantification. Fatty acids were also extracted from representative samples spiked with C_13:0_ internal standards (∼80% recovery) to assess cellular fatty acid detection.

### Lipid Profile Analysis by Thin Layer Chromatography and Subsequent Methyl Esterification (TLC-FAME Analysis)

Thin-layer chromatography (TLC) analysis was conducted as described previously (Vieler et al., 2007; Radakovits et al., 2011). Briefly, 10 ml of pelleted culture was resuspended in 400 μl MeOH and then sonicated for 10 min for cell lysis. Then 400 μl chloroform was added to solubilize lipids, followed by the addition of 400 μl water for phase separation. The organic layer containing lipids was transferred to new microcentrifuge tubes and dried under a N_2_ stream. Concentrated lipids were resuspended in 30 μl chloroform, then 4.0 μl of resuspended lipids were spotted onto HPTLC-HL normal phase, 150 mm silica gel plates (10 cm × 20 cm; Analtech, Newark, DE, USA) and developed in a TLC chamber. A series of lipid standards, which included monogalactosyldiacylglycerol (MGDG), digalactosyldiacylglycerol (DGDG) and sulfoquinovosyldiacyl glycerol (SQDG; Lipid Products, Nutfield, UK); phos phatidylcholine (PC), phosphatidylethanolamine (PE) and phosphatidylglycerol (PG; Avanti Polar Lipids, Inc., Alabaster, AL); cholesterol (Chol), palmitic acid (free fatty acid; FFA) and glyceryl trioleate (triacylglycerol; TAG; Sigma–Aldrich, St. Louis, MO, USA), were run concurrently to identify different lipid classes. The first eluent [(methyl acetate:isopropanol: chloroform:methanol:KCl (0.25%)] in a ratio of 25:25:25:10:4 (v/v/v/v/v), ran to a height of ∼5 cm from the origin. After drying, the plates were developed with a second eluent [hexane:diethylether:acetic acid in a ratio of 70:30:2 (v/v/v)] to a height of ∼8 cm from the origin. TLC plates were then sprayed with a 0.05% solution of primuline (TCI America, Portland, OR, USA) in acetone. Individual lipid bands were visualized under UV light at 365 nm. Following TLC separation, the individual lipid bands were marked and scraped from the TLC plates. The fatty esters in each lipid class were transesterified into FAMEs and analyzed by GC-FID, as described previously (Radakovits et al., 2011).

### Total RNA Extraction and Transcriptome Sequencing/Assembly

Total RNA was extracted from 20 ml of *Chaetoceros* GSL56 cells at stationary phase that were grown in f/2 medium at 3.5% salinity, supplemented with 1.06 × 10^-4^ M Na_2_SiO_3_. The plant RNA reagent (Invitrogen, Grand Island, NY, USA) was used to purify RNA from cells according to the manufacturer’s instructions. Genomic DNA was removed by DNase I (RNase free, Ambion, Grand Island, NY, USA) treatment, and subsequent RNA purification was carried out using the RNeasy MinElute Cleanup Kit (Qiagen, Germantown, MD, USA). Purified RNA was first quantified using a NanoDrop ND-1000 (Thermo scientific, Grand Island, NY, USA), and then more accurately measured using the QuantiT RiboGreen RNA assay kit (Invitrogen, Grand Island, NY, USA) with fluorescence detection.

Total RNA was sequenced by the National Center for Genomic Resources (NCGR, Santa Fe, NM, USA) as part of the Marine Microbial Eukaryote Transcriptome Project (Gordon and Betty Moore Foundation; Keeling et al., 2014). The RNA library was made with an insert size of ∼200 bp, using the TruSeq RNA Library preparation kit with poly-A+ selection. RNA was sequenced from both ends (paired-end reads 2 × 50 nt) using an Illumina Hi-seq 2000 (San Diego, CA, USA). The transcriptome dataset for *Chaetoceros* GSL56 is currently available through NCBI^1^.

Transcriptome assembly was conducted by NCGR using NCGR’s internal pipelines. Reads less than 25 bp after quality trimming were discarded, with the remaining reads assembled into contigs using ABySS (Simpson et al., 2009). All assembled contigs were subjected to gap closing using GapCloser v 1.10 (Li et al., 2008). To identify overlaps between contigs, the OLC (overlap layout consensus) assembler miraEST was used (Chevreux et al., 2004). BWA was used to align sequence reads back to assembled contigs (Li and Durbin, 2009). A final subset of contigs was created by filtering the contigs dataset by a minimal length of 150 bp. ESTScan with a Bacillariophyta scoring matrix was used to predict coding sequences (CDS) from the final subset of contigs (Iseli et al., 1999; Lottaz et al., 2003).

### Transcriptome Annotation and Molecular Phylogeny

All CDS sequences were aligned against the non-redundant (nr) protein databases at the National Center for Biotechnology Information (NCBI) using the BLASTx algorithm with an *E*-value cutoff of 10^-6^. The resulting top 10 blast hits were exported into Blast2Go software v 3.0.10 for functional annotation and statistical analysis (Conesa and Götz, 2008).

Transcripts putatively involved in fatty acid metabolism, including FAS and TAG synthesis, were reexamined. Representative sequences of each gene were downloaded from NCBI and used to search contig datasets to avoid assembly error. All identified transcripts were aligned against transcriptome reads in the NCBI Sequence Read Archive (SRA) database to check assembly integrity and coverage. To identify sub-classes of FAS genes, such as β-ketoacyl-ACP synthase I, II, and III (KASI, II, and III), alignment of translated amino acids sequences was conducted using MUSCLE 3.8.31 and phylogenetic trees were generated using the Phylogeny.fr program (Dereeper et al., 2008).

### KAS Complementation in *Synechococcus* 7002

Only one full length *KASIII* was annotated in the *Chaetoceros* GSL56 transcriptome (*KASIII* hereafter), which was amplified from *Chaetoceros* GSL56 cDNA using the primers listed in Supplementary Table S2. The amplified DNA fragment containing NdeI/HindIII restriction sites from the primers was inserted into a modified pNSI-cpcBA-YFP plasmid (Davies et al., 2014), containing a gentamicin resistance cassette (*aac*C1; Kovach et al., 1995) so that *KASIII* was positioned immediately after the *cpcBA* promoter (Xu et al., 2011) to form the new plasmid pNSI-cpcBA-gslKASIII-GentR. For homologous recombination, the *Synechococcus* 7002 *fabH* gene and flanking sequence (0.9 kb) was amplified from *Synechococcus* 7002 genomic DNA, and the resulting 2.0 kb DNA fragment inserted into plasmid pNSI-cpcBA-YFP-GentR by cloning in between the M13 forward and the M13 reverse sequences, generating plasmid pfabH. A 2.6 kb DNA fragment that included the *cpcBA* promoter, *KASIII* gene and the *aac*C1 marker was then amplified from pNSI-cpc-gslKASIII-GentR and inserted into pfabH to replace part (the first 500 bp) of the coding region of *fabH*, which is the target of gene disruption. The resulting plasmid, pfabH-cpcBA-gslKASIII-GentR, was used to transform *Synechococcus* 7002 for KASIII substitution assays. In order to minimize an imbalance between KASIII and other FAS enzymes in transgenic strains, another plasmid without the *cpcBA* promoter was created (plasmid pfabH-gslKASIII-GentR), in which *KASIII* expression was driven by the endogenous *Synechococcus* 7002 *fabH* promoter. This plasmid was constructed using designed primers (Supplementary Table S2), NEBuilder HiFi DNA assembly master mix (New England Biolabs Inc., MA) and Gibson cloning (Gibson et al., 2009) to avoid the use of restriction sites. A DNA fragment that contained the *KASIII* gene and the *aac*C1 marker gene was inserted into plasmid pfabH, replacing the first 500 bp of coding region of *fabH* at the start codon. DNA cloning was conducted in *Escherichia coli* DH5α cells. The genetic replacement of *fabH* with *KASIII* was conducted in a *Synechococcus* 7002 wild type strain and a lauric acid secreting (SA01) strain (Xu et al., 2013; Work et al., 2015).

To assess *in vivo* KASIII function, fully segregated transgenic strains were tested. Wild type and transgenic strains were first cultivated in 30 ml A+ medium in 125 ml flasks with the required antibiotic(s) (50 μg/ml gentamicin for SK01 and SK02, 50 μg/ml spectinomycin for SA01, 50 μg/ml gentamicin + 50 μg/ml spectinomycin for SAK01 and SAK02) at 37°C in an illuminated Percival incubator providing continuous illumination of ∼200 μmol PAR m^-2^ s^-1^ and aerated with 1% CO_2_/air. After 3–4 days of cultivation, all cultures were diluted in 50 ml fresh media (with antibiotics) in 250 ml flasks, and normalized to the same optical density (OD_730_ = 0.5). For fatty acid production, experimental cultures were cultivated at two different conditions: (A) room temperature, atmospheric CO_2_, and constant illumination at ∼80 μmol photons m^-2^ s^-1^ of PAR; and (B) 30°C, 1% CO_2_ and constant illumination at ∼80 μmol photons m^-2^ s^-1^ of PAR inside a growth chamber (Multitron, AJ125BC).

## Results

### *Chaetoceros* GSL56 Fatty Acid Profiles and Physiology

**FIGURE 1:**
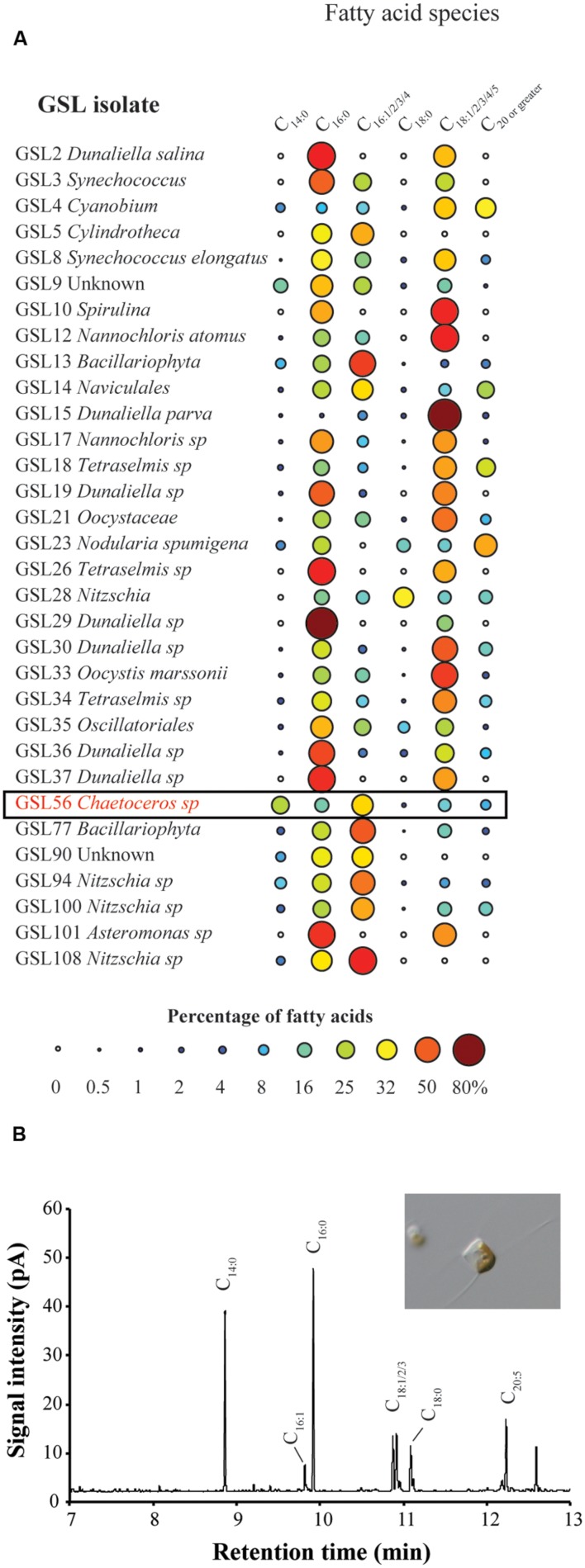
**Fatty acid screen of GSL culture collection **(A)** and representative GC-FID fatty acid methyl ester (FAME) profile derived from *Chaetoceros* GSL56 fatty acids **(B)**.** The size of each circle in **(A)** is proportional to the percentage of total fatty acid represented by the corresponding fatty acid species. Representative microscopic image from *Chaetoceros* GSL56 grown in f/2 medium is inset in **(B)**.

To identify organisms and enzymes capable of facilitating MCFA production in algae and cyanobacteria, we surveyed an algal collection isolated from GSL for organisms with natively high levels of MCFA (**Figure 1A**). Relative to the 42 halophilic algae that we isolated from this site, the ratio of C_14:0_ to other fatty acids was the highest in *Chaetoceros* GSL56 as determined by FAME analysis (**Figure 1**; **Table 1**). C_14:0_ fatty acid reached ∼20–35% of the total fatty acid content in this alga, depending on growth conditions and phases. Marine diatoms (e.g., *Chaetoceros*, *Thalassiosira*, and *Phaeodactylum*) tend to accumulate more C_14:0_ fatty acid (by percentage) relative to representative green algae [e.g., *Chlamydomonas* and *Chlorella* (**Table 1**)]. *Chaetoceros* GSL56 grew faster and reached higher cell numbers in medium containing 3.5% salinity, compared to lower and higher NaCl concentrations, 1.75 and 6% salinity, respectively (Supplementary Figure S1). Growth under different light intensities indicated that cells grew to a slightly higher density at lower light intensities (20 μmol PAR m^-2^ s^-1^ and 60 μmol PAR m^-2^ s^-1^) relative to a higher light intensity (120 μmol PAR m^-2^ s^-1^), where the final cell concentrations were decreased relative to cultures at lower light. Therefore, cells were cultivated in medium containing 3.5% salinity at 60 μmol PAR m^-2^ s^-1^, unless otherwise noted.

**Table 1:** Percentages of fatty acids in GSL56 relative to other indicated algal strains.

	Bacillariophyceae (diatoms)				Chlorophytes (green algae)	
		
	*Chaetoceros* GSL56^∗^	*Chaetoceros* sp. C256^1^	*Thalassiosira ps*. (Amursky)^2^	*Phaeodactylum tr*. NIVA-BAC2^3^	*Chlamydomonas re*. CC125^4^	*Chlorella vu*. UTEX 259^5^
**Saturated fatty acids**	
C_14:0_	26.0 (11.4)	18.8	12.4	9.5		2.0
C_16:0_	22.8 (4.9)	5.5	13.4	17.9	22.1	20.0
C_18:0_	2.1 (0.4)	1.5	2.5	0.6	2.4	1.0
Sum	50.9 (15.3)	25.8	28.3	28.0	24.5	23.0
**Unsaturated fatty acids**	
C_16:1/2/3/4_	25.1 (14.5)	43.1	33.7	28.0	9.1	26.0
C_18:1/2/3/4/5_	12.0 (7.0)	6.9	11.0	8.2	66.4	51.0
C_20:1/4_	3.6 (3.3)	3.0	-	2.4	-	-
C_20:5_ (EPA)	2.1 (1.8)	16.7	19.3	30.6	-	-
C_22:6_ (DHA)	0.1 (0.2)	0.8	2.2	0.2	-	-
Sum	42.9 (17.1)	70.5	66.2	69.3	75.5	77.0


*^∗^Fatty acids profiles were calculated from four biological samples at stationary phase, grown in f/2 medium with 3.5% salinity, 29°C. Standard deviations are shown in parentheses. ^1^Renaud et al., 1999; ^2^Zhukova, 2004; ^3^Patil et al., 2007; ^4^Moellering and Benning, 2010; ^5^Breuer et al., 2012. -, not detected.*

**FIGURE 2:**
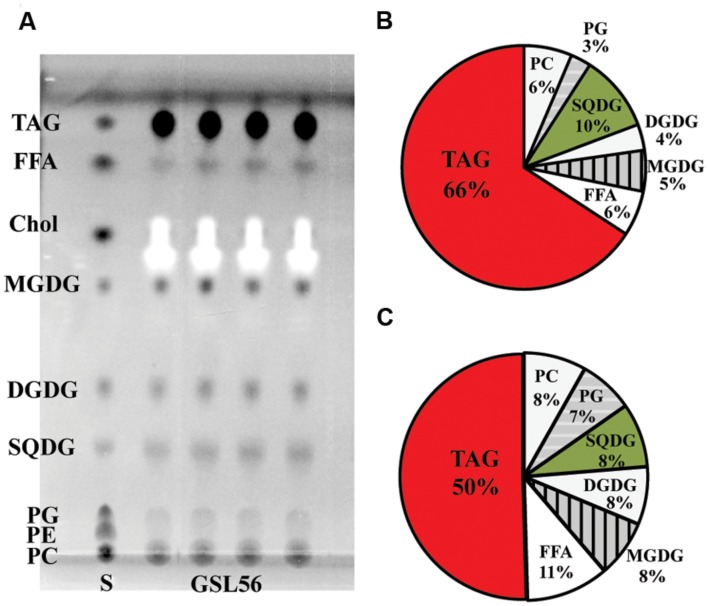
***Chaetoceros* GSL56 lipid classes separated by thin-layer chromatograpy **(A)**, C_14:0_ distribution within the lipid classes **(B)**, and the relative distribution of all lipids classes **(C)**.** Four lipid samples extracted from GSL56 cells were run concurrently with lipid standards (S) on TLC plates. Each data set represents two biological replicates. Abbreviations: Chol, cholesterol; DGDG, digalactosyldiacylglycerol; FFA, free fatty acid; MGDG, mono-galactosyldiacylglycerol; PC, phosphatidylcholine; PE, phosphatidylethanolamine; PG, phosphatidylglycerol; SQDG, sulfoquinovosyldiacylglycerol; TAG, triacylglycerol.

To further understand the biosynthesis and physiology of C_14:0_ fatty acid in *Chaetoceros* GSL56, specifically whether C_14:0_ accumulates predominately in membranes or storage products, lipids classes were extracted from cultures at stationary phase and subjected to TLC-FAME analysis (**Figure 2**). TLC analysis showed that the dominant lipid classes in *Chaetoceros* GSL56 include two phospholipids (PC and PG), SQDG, DGDG, MGDG, free fatty acids (FFAs), and triacylglyceride (TAG) (**Figure 2A**). The FFAs may represent artifacts of sample preparation. Sequential FAME analysis revealed that ∼66% of C_14:0_ fatty acid was stored in TAG and the second largest portion was in SQDG (∼10%; **Figure 2B**). Compared to total fatty acid distributions, about 50% of total fatty acids were found in TAG and 8% was in SQDG, demonstrating enrichment in these two lipids classes (**Figure 2C**).

### Transcriptome Analysis and Functional Annotation

To investigate the fatty acid biosynthetic pathway underpinning high C_14:0_ fatty acid content in *Chaetoceros* GSL56, we obtained a whole-cell transcriptome and assembled the data into expressed genes. Whole transcriptome sequencing was carried out using a cDNA library obtained from cells at stationary phase. A total of 36,745,292 raw reads were generated from sequencing the cDNA library using Illumina HiSeq 2000 (**Table 2**). The paired raw reads are available in the NCBI Sequence Read Archive (SRA) database with the accession number SRX551298. After trimming and cleaning sequences for adapters and primers, preprocessed sequences were assembled into 16,977 contiguous sequences (contigs) with sizes ranging from 150 to 18,638 bp. A subset of 14,914 contigs was predicted to contain protein CDS by ESTscan (150–17,385 nt in length, average = 1,720 nt, 14,088 unique), and 11,176 (∼80%) of these unique sequences had homologs when aligned against the nr database in NCBI using BlastX (*E*-value ≤ 10^-6^). The top five species contributing to predicted transcript annotation were *Thalassiosira pseudonana* (diatom, 16%)*, Phaeodactylum tricornutum* (diatom, 15%), *Ectocarpus siliculosus* (brown algae, 6%), *Phytophthora infestans* (oomycete, 4%) *and Aureococcus anophagefferens* (heterokont, 4%) (Supplementary Figure S2).

**Table 2:** Summary of *Chaetoceros* GSL56 transcriptome sequencing.

	Number of sequences
Reads assembled as contigs	16,977
Predicted coding sequences	14,914
Unique coding sequences	14,088
Unique coding sequences with BLAST hits	11,176
Sequences annotated with gene ontology (GO) terms	9,055
Sequences assigned with enzyme commission (EC)	1,638


The most represented annotations belonging to each GO category are listed in Supplementary Figure S2. Transcripts predicted to encode proteins in the molecular function category are more abundant than in the other two categories, cellular component and biological process. In the molecular function category, the GO term “protein binding” is the most represented, and in cellular component and biological process, the GO terms “integral component of membrane” and “oxidation-reduction process” are the most represented, respectively.

### Analysis of Fatty Acid Biosynthesis Pathway

**FIGURE 3:**
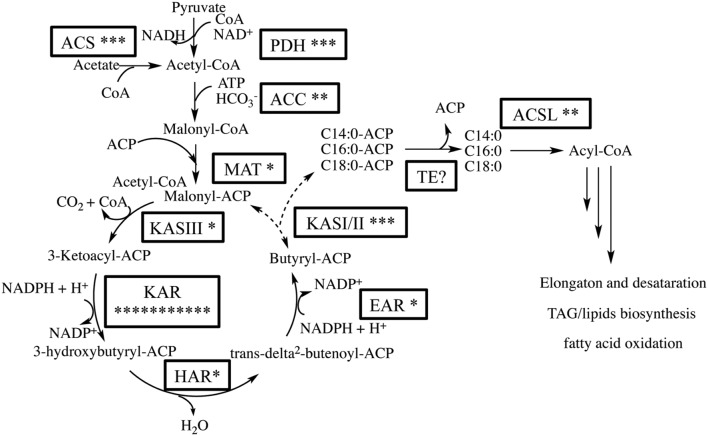
**Putative fatty acid synthesis (FAS) pathway in *Chaetoceros* GSL56 based on functional annotation.** The asterisks attached to the enzyme name indicate the putative number of isozymes encoded by genes present in the transcriptome. Enzyme abbreviations: ACC, acetyl-CoA carboxylase; ACS, acetyl-CoA synthetase; ACSL, long chain acyl-CoA synthase; EAR, enoyl-ACP reductase; TE, acyl-ACP thioesterase; HAR, hydroxyacyl-ACP dehydratase; KAR, β-ketoacyl-ACP reductase; KAS, β-ketoacyl-ACP synthase; MAT, malonyl-CoA-ACP transacylase; PDH, pyruvate dehydrogenase E1.

**FIGURE 4:**
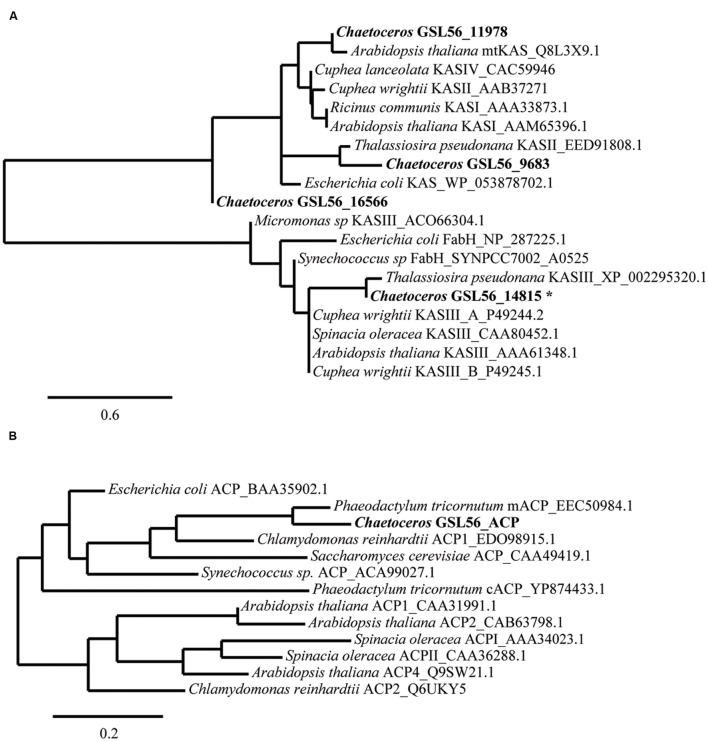
**Sequence similarities between *Chaetoceros* GSL56 β-ketoacyl-ACP synthase (KAS) **(A)** and acyl carrier proteins (ACP) **(B)** relative to indicated plant, bacteria, and algae representatives.** Scale bars represent the branch lengths: the number of amino acid substitutions per site. The asterisk indicates the sequence that was used in KASIII substitution assay.

We expected that the predominant control of MCFA chain lengths occurs in the fatty acid biosynthetic pathway since C_14:0_ is present in all lipids classes. Genes putatively involved in the *de novo* FAS pathway were first identified (**Figure 3**; Supplementary Table S3). Examination of gene isozymes with putative functions in fatty acid initiation and elongation indicates an underrepresentation of the genes in the upstream portion of this pathway, when compared to *Chlamydomonas reinhardtii* and two diatoms – *T. pseudonana and P. tricornutum* (Miller et al., 2010; Boyle et al., 2012; Dyhrman et al., 2012). In the transcript assembly, we could only identify a single gene encoding an acyl-carrier protein (ACP), which acts as a tether shuttling growing fatty acids to multiple components of the fatty acid synthase complex. The predicted polypeptide is 72% identical to an ACP from the diatom *P. tricornutum* (GenBank: EEC50984.1; **Figure 4B**). In both the *C. reinhardtii* and *T. pseudonana* transcriptomes, two ACPs with relatively high expression levels are found. Two putative monomeric acetyl-CoA carboxylase (ACCase) enzymes were identified in the *Chaetoceros* GSL56 transcriptome (transcript ID: 5820 and 12896) that have 82–85 and 72–76% similarities with representatives in *P. tricornutum* and *T. pseudonana*, respectively. Heteromeric ACCase enzymes that have dissociated enzymatic subunits were not identified, whereas they were identified in *C. reinhardtii*. The abundances of these two ACCase transcripts in *Chaetoceros* GSL56 was low as indicated by calculated reads per kilobase per million mapped reads (RPKM) values (13 and 0.4), in contrast to ACCase transcript abundance in *C. reinhardtii* (RPKM 246.5 and 250; Miller et al., 2010). It should be noted that a direct comparison of RPKM values between species may be compromised due to different genome sizes and different isozymes. Thus, we compared the RPKM of ACCase to other genes involved in the fatty acid biosynthetic pathway within the same species. Whereas the ACCase ranks high in the fatty acid biosynthesis pathway in *C. reinhardtii* (Miller et al., 2010; Lv et al., 2013), it is among the least abundant transcripts in *Chaetoceros* GSL56. In contrast to ACP and ACCase, the genes involved in fatty acid elongation reactions are more abundant in *Chaetoceros* GSL56 relative to *C. reinhardtii*. For example, 11 unique isozymes were identified for the 3-ketoacyl reductase (KAR) enzymes, with the highest RPKM value equal to 245, whereas in *C. reinhardtii* only one copy of this gene was found. Transcripts encoding hydroxyacyl-ACP dehydratase (HAR) and enoyl-ACP reductase (EAR) were also identified at high abundance (RPKM: 267 and 131, respectively).

Substrate specificity of some key enzymes in *Chaetoceros* GSL56 is likely to lead to C_14:0_ fatty acid accumulation. In plants, two enzymes, ketoacyl-ACP synthase (KASI/II, KASIII) and acyl-ACP TEs (FatA/B) typically show different enzymatic activities toward different fatty acid chain lengths, which contribute to fatty acid chain length determination (Jones et al., 1995; Leonard et al., 1998; Abbadi et al., 2000; Dehesh et al., 2001). Therefore, we initially attempted to identify genes encoding these two enzyme classes in *Chaetoceros* GSL56. To maximize gene identification, protein queries that consisted of homologous genes from diatoms and several other algae were conducted. Putative genes were then aligned with well-characterized genes for further annotation (**Figure 4**). There are four distinct transcripts annotated as KAS. Based on sequence alignments with KAS genes from plants and bacteria (**Figure 4A**), one transcript (14815) likely encodes a plant type KASIII enzyme, and it is not related to the other KAS enzymes. Transcript (9683) is predicted to be a KASII enzyme that is similar to KASII from *P. tricornutum*. Transcript 11978 has homology with plant type KASI/II/IV, while the fourth transcript (16566) has only tenuous KAS similarity. Unambiguous TE identification is complicated since there are often no clear homologs to the conical plant-type enzymes found in diatoms. As expected, a homolog of this type of an acyl-ACP TE was not identified in *Chaetoceros* GSL56 (data not shown); however, genes encoding putative thioester hydrolyzing enzymes were found (transcripts: 2647, 3750, 4370, 9448, and 9539).

### KASIII in *Chaetoceros* GSL56 Substitutes for FabH in *Synechococcus* sp. PCC7002

**FIGURE 5:**
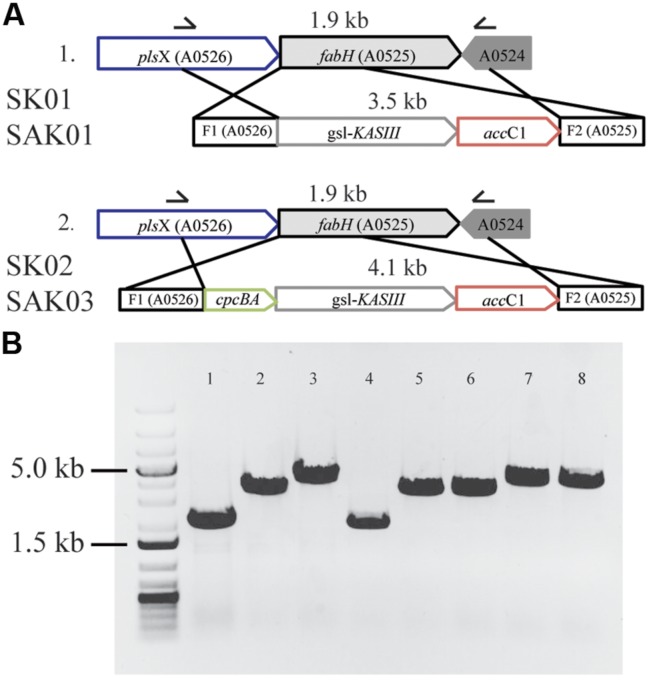
**Substitution of KASIII from *Chaetoceros* GSL56 replacing FabH in *Synechococcus* 7002.** **(A)** Schematic of homologous recombination for *KASIII* gene complementation. **(B)** Whole cell PCR with gene specific primers demonstrating the integration of recombinant genes in *Synechococcus* 7002 transformants. From left to right: molecular size marker: 1 kb plus DNA ladder; 1: *Synechococcus* 7002 wildtype strain; 2: SK01; 3: SK02; 4: SA01; 5–6: two different lines of SAK01; 7–8: two different lines of SAK03.

As shown above, *Chaetoceros* GSL56 effectively accumulates high levels of MCFA. However, the data also demonstrate that this strain has poor growth and biomass productivity metrics (Supplementary Figure S1). We therefore examined whether enzymatic “parts” from *Chaetoceros* GSL56 could be used in a biofuel “chassis” organism (*Synechococcus* 7002) to improve a biofuel phenotype, in this case MCFA synthesis and secretion. First, each of the genes potentially encoding thioester hydrolyzing enzymes were cloned and transformed into *Synechococcus* 7002 concomitantly with *fadD* disruption to enable fatty acid secretion, an approach that was successfully used previously for genuine thioesterase enzymes (Work et al., 2015); however, none of the encoded enzymes showed acyl-ACP TE activity in *Synechococcus* 7002 (data not shown). We then explored whether expression of KASIII (functional homolog of endogenous FabH) could influence FAS in this cyanobacterium. A recent report concluded that FabH is the rate-limiting step in FAS in *Synechococcus* 7002 (Kuo and Khosla, 2014). Plasmids were designed for concurrently knocking out the native *Synechococcus* 7002 *fabH* gene while inserting *KASIII* from *Chaetoceros* GSL56 in its place. Enzyme expression was driven either at native levels by the endogenous *fabH* promoter (SK01) or at very high levels by the *cpcBA* promoter (SK02; Xu et al., 2011). Fully segregated transgenic strains were obtained through repeating streaking of single colonies on A+ plates containing the required antibiotics (**Figure 5**). Controls using a plasmid that had yellow fluorescent protein instead of *KASIII* from *Chaetoceros* GSL56 were also generated, but these mutants were not able to reach homoplasmy, indicating that FabH (or a functional replacement) is essential for *Synechococcus* 7002 viability. Relative to the wildtype *Synechococcus* 7002, the two transgenic strains expressing KASIII (SK01 and SK02) showed no major differences in either fatty acid profiles or total fatty acid production when transformed into the wildtype background (data not shown). We then transformed a *Synechococcus* 7002 strain SA01 (Work et al., 2015) that is able to secrete lauric acid (C_12:0_) due to the expression of the medium chain acyl-ACP TE from *Umbellularia californica* (*UcFatB*; **Table 3**). As the SA01 strain is already able to secrete FFAs, we probed whether enhanced MCFA synthesis would occur when the rate-limiting endogenous FabH was replaced with the *Chaetoceros* GSL56 KASIII. KASIII substitution/overexpression in other systems has been shown to influence fatty acid profiles/yields, putatively by changing the rate of fatty acid initiation relative to downstream enzyme activities, and/or by catalyzing elongation reactions for short chain (but not long chain) acyl-ACP substrates (Abbadi et al., 2000; Dehesh et al., 2001; González-Mellado et al., 2010).

**Table 3:** *Synechococcus* 7002 strain used and transgenic strain constructed in this study.

Strain	Description	Reference
***Synechococcus* 7002**	**Wild type**	
SK01	*Synechococcus* 7002 with gene knockout of *fabH* and expression of *Chaetoceros* GSL56 *KASIII* (driven by native *fabH* promoter)	This study
SK02	*Synechococcus* 7002 with gene knockout of *fabH* and expression of *Chaetoceros* GSL56 *KASIII* (driven by *cpcBA* promoter^a^)	This study
SA01	*Synechococcus* 7002 with gene knockout of acyl–acyl carrier protein synthetase (Δ*aas*) and expression of *Umbellularia californica* acyl-ACP thioesterase (driven by *cpcBA* promoter^a^)	Work et al., 2015
SAK01	SA01 with gene knockout of *fabH* and expression of *Chaetoceros* GSL56 *KASIII* (driven by native *fabH* promoter)	This study
SAK03	SA01 with gene knockout of *fabH* and expression of *Chaetoceros* GSL56 *KASIII* (driven by *cpcBA* promoter^a^)	This study


*^a^Xu et al. (2011).*

### Enhanced Lauric Acid Production in Transgenic Strains

**FIGURE 6:**
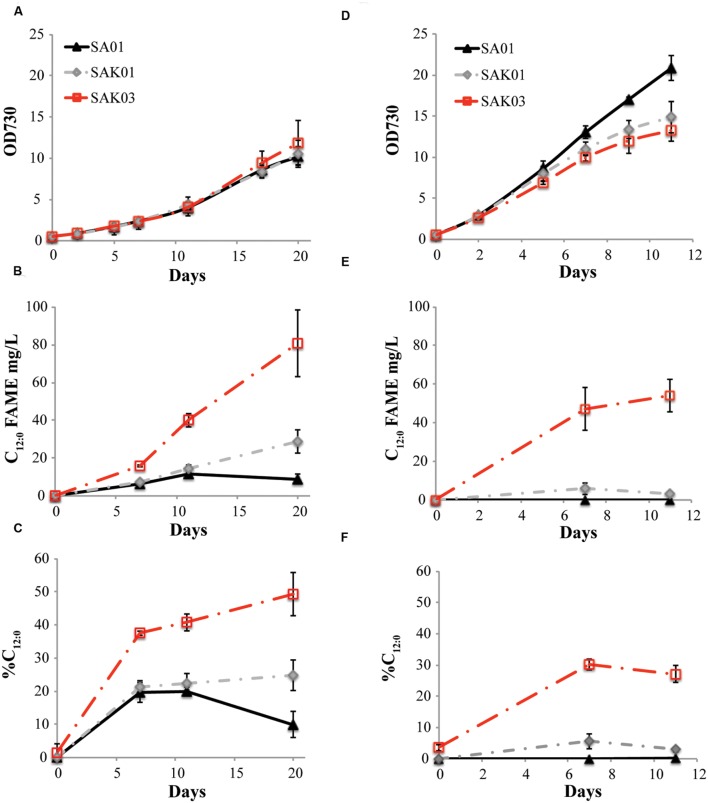
**Comparison of C_12:0_ fatty acid production using two different growth conditions.** **(A,D)** optical density of cultures measured at 730 nm, **(B,E)** C_12:0_ fatty acid production, and **(C,F)** percentage of C_12:0_ fatty acid relative to total fatty acids. Growth condition #1 **(A–C)**: room temperature, atmospheric CO_2_; growth condition #2 **(D–F)**, 30°C, 1% CO_2_. Data represent three independent biological replicates.

**FIGURE 7:**
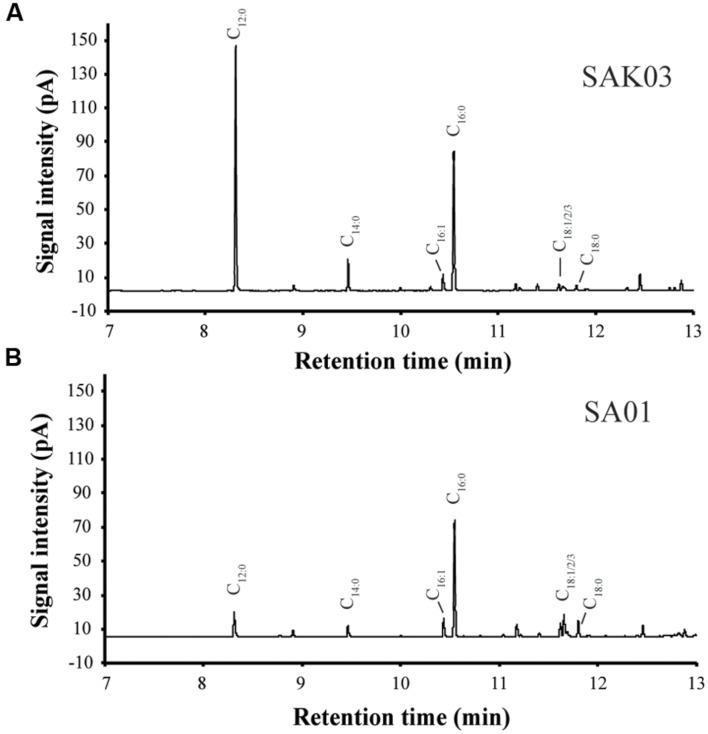
**Representative GC-FID chromatograms of FAMEs from the *Synechococcus* 7002 mutants SAK03 **(A)** and SA01 **(B)**.** FAMEs were extracted from batch cultures grown at room temperature and harvested on day 20.

When FabH was replaced by the *Chaetoceros* GSL56 KASIII enzyme in the *Synechococcus* 7002 SA01 background, both new transgenic strains (SAK01 and SAK03) showed enhanced levels of C_12:0_ fatty acid production/secretion relative to SA01 (**Figure 6**; **Table 4**). An increase was observed under both of the two different culturing conditions tested. For cells grown at room temperature without CO_2_ augmentation (growth condition #1), SA01, SAK01, and SAK03 all grew similarly (**Figure 6A**). Despite no noticeable growth differences between SA01 and the *KASIII* expressing mutants, C_12:0_ fatty acid accumulated at much higher levels per ml of culture and in the relative percentage of all fatty acids. The enhancement ranged from 1.1 to 5-fold depending on growth phases and the promoters used. In SA01, the highest levels of C_12:0_ accumulation occurred at day 11 (11.5 mg/L, 20% of total fatty acids) and started to slightly decrease thereafter (**Figures 6B,C**). However, in the *KASIII* expressing strains, the C_12:0_ fatty acid levels continue to show increases even at day 20. The relative percentages of C_12:0_ fatty acid also continued to increase during the experimental period. We also tested SA01, SAK01, and SAK03 at higher temperature (30°C) and supplemented with 1% CO_2_ (growth condition #2). All strains grew faster than in growth condition #1. Under these conditions, the transgenic strains (SAK01/03) showed defective growth relative to SA01 (**Figure 6D**); and unexpectedly, all strains showed decreased C_12:0_ fatty acid productivity and a lower percentage of C_12:0_ than in growth condition #1 (**Figures 6E,F**). The highest amount of C_12:0_ (54 mg/L, 30% of all fatty acids) was produced from SAK03 with *KASIII* expression driven by the *cpcBA* promoter. In sum, expression of KASIII in *Synechococcus* 7002 and co-expression of a medium chain specific thioesterase enhances MCFA synthesis (C_12:0_, **Figure 7**) in *Synechococcus* 7002 under the growth conditions tested.

**Table 4:** Fatty acid profiles of *Synechococcus* 7002 wildtype strain and transgenic strains from *KASIII* substitution assays sampled at day 11.

	Total FA	Relative percentage of individual fatty acid					
		
	mg/L	C_12:0_	C_14:0_	C_16:0_	C_16:1_	C_18:0_	C_18:N_^a^
7002	61.08 (12.66)	0.00 (0.00)	0.45 (0.04)	47.35 (2.02)	17.22 (1.78)	1.25 (0.29)	33.73 (2.43)
SA01	53.99 (7.84)	16.98 (6.07)	2.14 (0.32)	38.45 (3.18)	14.00 (2.25)	3.89 (0.34)	24.54 (2.97)
SAK01	63.76 (8.18)	22.38 (3.07)	2.57 (0.49)	35.44 (1.09)	13.21 (1.60)	3.21 (0.58)	23.17 (3.97)
SAK03	98.46 (5.52)	40.75 (2.53)	3.95 (0.15)	28.61 (1.52)	12.77 (0.40)	1.06 (0.15)	12.79 (2.42)


*Samples were cultivated at room temperature, 0.04% CO_2_ (condition 1); data are average of three independent biological replicates, and standard deviations are shown in parentheses. ^a^C_18:N_ includes C_18:1_, C_18:2_, and C_18:3_.*

## Discussion

Medium chain fatty acids are desirable in both the fuel and chemical industries (Battey et al., 1989). However, large quantities of MCFAs are only available in some specific oilseeds, such as coconut and *Cuphea*. Expression of MCFA specific enzymes can lead to the accumulation of shorter chain fatty acids in crop plants, providing a way to increase MCFA productivity (Voelker et al., 1992). In this study, we explored FAS in a halophilic alga (*Chaetoceros* GSL56) that is enriched for MCFA, and used whole cell transcriptome sequencing to identify genes encoding putative FAS enzymes. Significantly, we identified a gene encoding a KASIII enzyme that when expressed in *Synechococcus* 7002 is able to functionally replace the native cyanobacterial FabH enzyme, and when co-expressed in a C_12:0_ MCFA secreting strain results in increased levels of MCFA synthesis.

*Chaetoceros* GSL56 primarily synthesizes C_14:0_, C_16:0_, C_18:0_, C_18:N_, and C_20:5_ fatty acids that are differentially incorporated into all lipid classes (**Table 1**; **Figures 1** and **2**). Depending on growth conditions, saturated C_14:0_ fatty acids range from ∼15–40% of all fatty acids in *Chaetoceros* GSL56 which is amongst the highest native C_14:0_ levels that we have observed in an alga (**Figure 1A**). Additional marine algae have also been documented to contain MCFA, primarily within the Bacillariophyta and Haptophyta phyla (Chen et al., 2007; Guihéneuf et al., 2010). Analysis of individual lipid classes in *Chaetoceros* GSL56 revealed that MCFAs are preferentially incorporated into TAG (**Figure 2**; Supplementary Table S1), which is consistent with the MCFA profiles in plant seeds and the other diatom species (Battey and Ohlrogge, 1989; Alonso et al., 2000; Chen et al., 2007). In contrast to neutral oil droplets, the phospholipids and galactolipids, which are used as membrane constituents, are enriched in long chain fatty acids (C_16-20_).

Although *Chaetoceros* GSL56 has a promising MCFA phenotype, this alga has poor growth metrics relative to biotechnologically relevant strains. We therefore identified enzymes in the MCFA biosynthetic pathway that could be used as “parts” in more biotechnologically promising strains. *Synechococcus* 7002 is a cyanobacterium with among the fastest growth rates of any PSM, and has become the recent focus of several biotechnology efforts (McNeely et al., 2010; Mendez-Perez et al., 2011; Davies et al., 2014; Work et al., 2015). We therefore transferred selected components of the *Chaetoceros* GSL56 FAS machinery into *Synechococcus* 7002 to (i) verify functional annotations and (ii) determine whether enzymes from *Chaetoceros* GSL56 could be expressed in a cyanobacterium to increase MCFA synthesis. Based on precedence in plant studies, (Voelker et al., 1992; Dehesh, 2001), we targeted substrate specific enzymes, such as acyl-ACP TEs and β-ketoacyl ACP synthase (KAS), to influence MCFA synthesis (Voelker et al., 1992; Leonard et al., 1998).

Initially, we probed whether five genes encoding putative thioester hydrolysis enzymes contained acyl-ACP TE activity that could be used to produce MCFAs in *Synechococcus* 7002. Expression of each of these genes individually while concurrently knocking out the gene encoding the fatty acid recycling enzyme FadD (Kaczmarzyk and Fulda, 2010; Liu et al., 2011; Ruffing, 2014; Work et al., 2015), did not result in the production of any MCFA in *Synechococcus* 7002. This may be because the enzymes tested were not genuine acyl-ACP TEs, or because they did not fold into functional enzymes in *Synechococcus* 7002. We then targeted the KAS enzyme family. The β-ketoacyl ACP synthase III (KASIII) functionally replaced the FabH enzyme in *Synechococcus* 7002, confirming the annotation of KASIII as a genuine β-ketoacyl ACP synthase III. Furthermore, expression of KASIII in a *Synechococcus* 7002 strain that coexpresses a plant-sourced medium chain specific thioesterase (*UcfatB*) produced up to 40% of total fatty acid as lauric acid (SAK03), which is approximately four times more than corresponding strains expressing the native FabH enzyme. Enhanced lauric acid production in transgenic strains may be due to, (i) an increased acyl-ACP pool size since *fabH* is the rate-limiting enzyme of fatty acid synthase in *Synechococcus* 7002 (Kuo and Khosla, 2014); (ii) modified overall FAS rate that better matches thioesterase activity (Dehesh et al., 2001); and/or, (iii) concentrated short to medium chain acyl-ACPs pools caused by KASIII (Abbadi et al., 2000). The exact mechanism of KASIII enhancement, and testing whether KASIII participates in MCFA synthesis in *Chaetoceros* GSL56, is the subject of further investigation.

The euryhaline cyanobacterium *Synechococcus* 7002, is a particularly promising host for FFA production, because it shows a unique high tolerance to FFAs (Ruffing, 2014). However, the total metabolic flux to FAS represents a small portion (∼5–10%) of cell dry weight, indicating evolutionary limitations to fatty acid productivities in this host (Work et al., 2015). Replacing FabH, which is the kinetically rate-limiting enzyme in FAS, with KASIII did not improve fatty acid production when transformed into the wild-type host (data not shown), suggesting that downstream elements that traffic fatty acids to membrane lipids may now be rate limiting. However, the expression of acyl-ACP thioesterase allows cleavage of MCFA from acyl-ACP, and has been reported to increase malonyl-ACP turnover rates and reduce the inhibition of acyl-ACP in other systems (Magnuson et al., 1993). Transgenic strains expressing KASIII and thioesterase (SAK01 and SAK03) accumulated higher amounts of C_12:0_ and C_14:0_ fatty acids, relative to their parental strain SA01, and these increases are consistent with *UcfatB* hydrolytic activity (Voelker and Davies, 1994). Levels of MCFA are influenced by promoter strength, indicating a correlation between *KASIII* expression levels and MCFA production.

It is still undetermined how *Chaetoceros* GSL56 regulates C_14_ fatty acid production as we were unable to identify an acyl-ACP TE with C_14:0_ specificity in this alga, which is consistent with other studies in red algae and diatoms (Beld et al., 2014). Future studies of the role of KASIII on medium chain FAS, as well as the substrate specificities of other enzymes, such as acyltransferase, will be important in understanding fatty acid chain length regulation in this and other diatoms.

In future research, we also intend to explore whether, the expression of other eukaryotic FAS enzymes in *Synechococcus* 7002, which typically has less than 10% of its biomass in fatty acyl lipids, results in further yield improvements. It is possible that these eukaryotic enzymes, which did not evolve under the regulatory mechanisms used by cyanobacteria, remain active outside of the metabolic context that evolved to limit FAS in cyanobacteria such as *Synechococcus* 7002.

## Author contributions

HG designed and transformed *Synechococcus* 7002 strains, performed batch experiments and the majority of biochemical assays, and compiled this manuscript. RJ participated in GSL strain screening, generated data in **Figure 1** and revised this manuscript. FD participated in designing transformation vectors and revised this manuscript. LS and PS developed methods for extracting and quantitating internal standards. MP provided the conception of this work, oversaw and edited this manuscript. All authors revised the manuscript for intellectual content.

## Conflict of Interest Statement

The authors declare that the research was conducted in the absence of any commercial or financial relationships that could be construed as a potential conflict of interest.

## Footnotes

1 http://www.ncbi.nlm.nih.gov/bioproject/?term$=$PRJNA231566
