# Supplementary material for: Modulation of Medium-Chain Fatty Acid Synthesis in Synechococcus sp. PCC 7002 by Replacing FabH with a Chaetoceros Ketoacyl-ACP Synthase
Source: Front Plant Sci. 2016 May 26;7:690. doi: 10.3389/fpls.2016.00690 (PMC4880568; doi:10.3389/fpls.2016.00690)
Supplement: Supplementary file 1 [file Data_Sheet_1.DOCX]

**Figure S1. Effects of salinity (A) and light intensity (B) on cell growth of *Chaetoceros* GSL56.** Each data point is the average of 2 biological replicates.

**Figure S2. Functional annotation of GSL56 transcriptome, A) species distribution of top BLAST matches, and B) most represented GO terms in each of the indicated categories: cellular component, biological process and molecular function.**

**Table S1. Relative percentages of all fatty acids in individual lipid classes found in *Chaetoceros* GSL56.** Each data represents 2 biological replicates.

|  | Total lipids | | Percentage of different fatty acids | | | | | |
| --- | --- | --- | --- | --- | --- | --- | --- | --- |
|  | mg/L | C14:0 | | C16:0 | C16:1 | C18:0 | C18:n | > C20 |
| PC | 14.7 | 12.6 | | 25.9 | 0.8 | 10.1 | 33.5 | 13.5 |
| PG | 12.8 | 6.1 | | 26.1 | 1.0 | 11.4 | 38.9 | 12.2 |
| SQDG | 15.1 | 19.5 | | 24.4 | 1.1 | 10.2 | 31.7 | 9.3 |
| DGDG | 13.6 | 7.4 | | 21.5 | 1.1 | 13.1 | 37.3 | 13.6 |
| MGDG | 13.4 | 11.7 | | 22.5 | 1.3 | 9.9 | 36.1 | 14.6 |
| FFA | 19.4 | 8.9 | | 25.7 | 0.9 | 17.7 | 26.5 | 9.2 |
| TAG | 90.5 | 21.0 | | 18.6 | 34.5 | 2.1 | 10.5 | 10.8 |

**Table S2. Primers designed for *Synechococcus* 7002 transformations.** Introduced restriction sites are capitalized.

| gsl-*KASIII* gene amplification | |
| --- | --- |
| 14815_NdeI/HindIII | 5’-gcgtataaCATATGatggtaaccactggacctc-3’ |
|  | 5’-cccAAGCTTtcagatggaagatgactttcctc-3’ |
| pNSI-cpcBA-gslKASIII-GentR assembly | |
| pAQ1Ex_NdeI/HindIII | 5’-cccAAGCTTaacgagggcggtgctttgg-3’ |
|  | 5’-gcgtataaCATATGgcagacaattctacatcatcatc-3’ |
| pfabH assembly | |
| 7002_*fab*H_NdeI/BamHI | 5’-gcgtataaCATATGcattgatcggcctgcaattgg-3’ |
|  | 5’-actGGATCCctggcattggcttgatttggg-3’ |
| pNSI-cpcBA-YFP-NdeI/BamHI | 5’-gcgtataaCATATGgcttgagtattctatagtatcacc-3’ |
|  | 5’-actGGATCCcccaattcgccctatagtgag-3’ |
| pfabH-cpcBA-gslKASIII-GentR assembly | |
| pAQ1Ex01_SpeI/XhoI | 5’-gACTAGTgatgatgatgtagaattgtctgc-3’ |
|  | 5’-atcCTCGAGcgttgtgacaatttaccgaac-3’ |
| pAQ1Ex02_XhoI/SpeI | 5’-catCTCGAGcgtggtttgtcagagcagtcc-3’ |
|  | 5’-gACTAGTtgctcgactcccttccttcc-3’ |
| pfabH-gslKASIII-GentR assembly (Gibson assembly) | |
| F1-fwd | 5’catctccgaggaaggaagggagtcgagcaatggtaaccactggacctctcaaatgtcg-3’ |
| F2-fwd | 5’ggagttgttcggtaaattgtcacaacgccgccgtggtttgtcagag  cagtccccagga-3’ |
| F1-rev | 5’cgacatttgagaggtccagtggttaccattgctcgactcccttccttc  ctcggagatg-3’ |
| F2-rev | 5’tcctggggactgctctgacaaaccacggcggcgttgtgacaatttaccgaacaactcc-3’ |

**Table S3. Putative genes involved in fatty acid metabolism pathways found in the *Chaetoceros* GSL56 transcriptome assembly.**

| Enzyme | Symbol | EC number | Transcript ID |
| --- | --- | --- | --- |
| Acetyl-CoA synthesis |  |  |  |
| Pyruvate dehydrogenase | PDH | 1.2.4.1 | 5434 |
|  |  |  | 7580 |
|  |  |  | 11878 |
|  |  |  |  |
| Acetyl-CoA synthetase | ACS | 6.2.1.1 | 6778 |
|  |  |  | 14456 |
|  |  |  | 634 |
| Fatty acid biosynthesis |  |  |  |
| Acyl carrier protein | ACP |  | 5527 (2077:2421) |
| Biotin carboxylase | BC | 6.3.1.14 | 6554 |
| Acetyl-CoA carboxylase | ACC | 6.4.1.2 | 5820 |
|  |  |  | 12896 |
| Malonyl-CoA-ACP transacylase | MAT | 2.3.1.39 | 6099 |
| Beta-ketoacyl-ACP synthase I/II | KASI/II | 2.3.1.41, 2.3.1.179 | 16566 |
|  |  |  | 9683 |
|  |  |  | 11978 |
| Beta-ketoacyl-ACP synthase III | KAS III | 2.3.1.180 | 14815 |
| Beta-ketoacyl-ACP reductase | KAR | 1.1.1.100 | 12293 |
|  |  |  | 14555 |
|  |  |  | 10617 |
|  |  |  | 6515 |
|  |  |  | 11936 |
|  |  |  | 6307 |
|  |  |  | 4112 |
|  |  |  | 2376 |
|  |  |  | 7277 |
|  |  |  | 7111 |
| Hydroxyacyl-ACP dehydratase | HAR | 4.2.1.- | 1208 |
| Enoyl-ACP reductase | EAR | 1.3.1.9 | 12754 |
| Acyl-ACP thioesterase | FAT | 3.1.2.- | - |
| Palmitoyl-protein thioesterase | PPT | 3.1.2.22 | 9448 |
| Fatty acid elongation |  |  |  |
| Hydroxyacyl-CoA dehydrogenase | CHAD | 1.1.1.35 | 5539 |
|  |  |  | 2303 |
| Enoyl-CoA hydratase | ECH | 4.2.17 | 1942 |
|  |  |  | 2303 |
| Trans-2-enoyl-CoA reductase | TER | 1.3.1.38 | 11268 |
| Long chain fatty acid elongase | LKCS |  | 7180 |
| Very long chain fatty acid elongase | VLKCS | 2.3.1.199 | 12648 |
| Fatty acid desaturation |  |  |  |
| Fatty acid desaturase | FADS | 1.14.19 | 1650 |
| Δ5 fatty acid desaturase | DES-5 | 1.14.19 | 1655 |
| Δ6 fatty acid desaturase | DES-6 | 1.14.19 | 11960 |
| Δ9 acyl-ACP desaturase | AAD | 1.14.19.2 | 5341 |
| Δ12 fatty acid desaturase | DES-12 | 1.14.19.6 | 6631 |
|  |  |  | 4549 |
| Δ15 (ω3) fatty acid desaturase | DES-15 | 1.14.19- | 5304 |
| TAG synthesis |  |  |  |
| Acyl-CoA: glycerol-3-phophate acyltransferase | GPAT | 2.3.1.15 | 12510 |
|  |  |  | 14578 |
|  |  |  | 14651 |
|  |  |  | 2402 |
| Acyl-CoA:lysophosphatidate acyltransferase | LPAAT | 2.3.1.51 | 7438 |
| Acyl-CoA:diacylglycerol acyltransferase | DGAT | 2.3.1.20 | 2397 |
|  |  |  | 7092 |
|  |  |  | 3140 |
|  |  |  | 3195 |
|  |  |  | 4825 |
| Phospholipid:diacylglycerol acyltransferase | PDAT | 2.3.1.158 | 9601 |
| FA catabolism |  |  |  |
| Long chain acyl-CoA synthetase | ACSL | 6.2.13 | 5928 |
|  |  |  | 4327 |
| Acyl-CoA oxidase | ACX | 1.3.3.6 | 1502 |
|  |  |  | 5782 |
| Acyl-CoA dehydrogenase | ACADM | 1.3.8- | 5752 |
|  |  |  | 1502 |
|  |  |  | 8114 |
|  |  |  | 3369 |
| Enoyl-CoA hydratase | ECH | 4.2.17 | 1942 |
|  |  |  | 2303 |
| 3-hydroxyacyl-CoA dehydrogenase | CHAD | 1.1.1.35 | 5539 |
|  |  |  | 2303 |
| Acyl-CoA acetyltransferase | ACT | 2.3.1.9 | 5269 |
|  |  |  | 10035 |
| Enoyl-CoA isomerase | ECI | 5.3.3.8 | 2303 |
| dienoyl-CoA reductase |  |  | 8143 |
| Alcohol dehydrogenase | ADH | 1.1.1.1 | 549 |
| Aldehyde dehydrogenase | ALDH | 1.2.1.3 | 1619 |
|  |  |  | 3818 |
|  |  |  | 8812 |
|  |  |  | 11522 |
